# Progressive Improvement in 5-Year Survival Rates for Extremity Soft Tissue Sarcomas from 1999 to 2019

**DOI:** 10.1155/2024/8880609

**Published:** 2024-02-19

**Authors:** Ryley K. Zastrow, Mohyeddine El Sayed, Christa L. LiBrizzi, Andrew J. Jacobs, Adam S. Levin

**Affiliations:** ^1^Department of Orthopaedic Surgery, The Johns Hopkins Hospital, Baltimore, MD, USA; ^2^American University of Beirut, Beirut, Lebanon; ^3^CityMD Sayville Urgent Care, Sayville, NY, USA

## Abstract

**Background:**

Extremity soft-tissue sarcoma (ESTS) is a group of rare, heterogeneous malignancies. Previous studies have demonstrated a progressive improvement in 5-year survival rate over time, but recent trends are unknown. Therefore, this study aimed to provide an update on the clinical characteristics and 5-year survival rate of ESTS from 1999 to 2019.

**Methods:**

This retrospective cohort study used the Surveillance, Epidemiology, and End Results (SEER) database. Overall, 5,654 patients over the age of 15 years with primary ESTS diagnosed between 1999 and 2019 were included. Data on patient demographics, clinical characteristics, and survival were extracted. Patients were grouped by year of diagnosis: 1999–2005, 2006–2012, and 2013–2019. Kaplan–Meier and Cox proportional hazards regression analyses were performed.

**Results:**

ESTS occurred primarily in the lower extremity (76.1%) and was frequently grade III (58.3%), >5 cm in size (69.9%), and without metastasis (77.9%) at diagnosis. Furthermore, there was a significant increase in the proportion of patients over age 60 (*p* < 0.001) and without metastasis (*p* < 0.001) over the study period. The 5-year survival rate successively improved, from 47% in 1999–2005, to 61% in 2006–2012, to 78% in 2013–2019. Similarly, in multivariate analysis, the mortality rate progressively declined from a hazard ratio (HR) of 3.4 in 1999–2005 to an HR of 2.1 in 2006–2012, with the 2013–2019 group having the best overall survival (*p* < 0.001). Age, tumor size, grade, and metastasis were negative prognostic factors for survival; radiation and surgery were positive prognostic factors.

**Conclusions:**

The 5-year overall survival rate for ESTS progressively improved over the 20-year study period, perhaps due to an increasing proportion of older patients diagnosed with local disease. These findings may also be related to earlier detection or more effective treatment over the study period.

## 1. Introduction

Extremity soft tissue sarcoma (ESTS) comprises a group of rare, heterogeneous tumors of mesenchymal origin with a propensity for metastasis [1]. The most common histologic subtypes include undifferentiated pleiomorphic sarcoma, leiomyosarcoma, and liposarcoma [2]. The cornerstone of treatment is limb-preserving wide surgical resection, often coupled with radiation therapy, to reduce local recurrence [3]. The role of chemotherapy remains uncertain given the rarity and histologic variability of ESTS but is typically utilized for large, high-grade tumors or metastatic disease [4].

Several patient and clinical factors have been correlated with ESTS prognosis, including patient age, tumor size, and tumor grade [5–7]. Previous studies [5–6] have reported a 5-year survival rate of 50% to 56%, with a study by Jacobs et al. [7] demonstrating progressive improvement in survival from 28% to 62% from 1991 to 2010. However, the literature is limited with respect to more recent trends in ESTS survival. Therefore, this study aims to provide an update on the clinical characteristics and 5-year survival rate of ESTS from 1999 to 2019. We hypothesized that there would be stepwise improvement in survival rates over the study period.

## 2. Methods

This retrospective cohort study utilized the Surveillance, Epidemiology, and End Results (SEER) database, developed by the National Cancer Institute, which contains population-based cancer incidence and survival rates. Patients over the age of 15 diagnosed with primary ESTS from 1999 to 2019 were included. Patients with incomplete clinical data on tumor size, grade, metastasis, or treatment were included in incidence calculations but were excluded from all other analyses.

Data on patient demographics, clinical characteristics, and survival time were extracted. Patient demographics included sex, age (<30, 30–59, or ≥60), race (Caucasian, African American, or other), ethnicity (Hispanic or non-Hispanic), and marital status (married, single, other, or unknown). Clinical characteristics included year of diagnosis, tumor location (upper or lower extremity), size (<5, 5–10, or >10 cm), grade (I, II, or III), histologic type based upon ICD-O-3 codes (fibromatous, myxomatous, lipomatous, myomatous, synovial, not otherwise specified, or other), and initial treatment (surgery, radiation, and/or chemotherapy).

Patients were grouped by year of diagnosis for comparison: 1999–2005, 2006–2012, and 2013–2019. Incidence rates were age-adjusted to the 2000 United States standard population and calculated with SEER*∗*Stat (version 8.4; National Cancer Institute, Bethesda, MD). Annual percent change was tabulated via weighted least-squares method with the Tiwari modification for confidence intervals. Chi-square tests were used to compare patient and clinical characteristics between time periods, and Kaplan–Meier curves were used to compare survival rates. The effects of categorical and continuous variables on survival were assessed via log-rank test and Cox proportional hazards regression, respectively. Finally, Cox proportional hazards multivariate regression was performed with variables significantly associated with survival in univariate analyses. All statistical analyses were performed with IBM SPSS Statistics, version 22.0 (Armonk, NY: IBM Corp; 2013). A *p* value <0.05 was considered statistically significant.

## 3. Results

**Figure 1:**
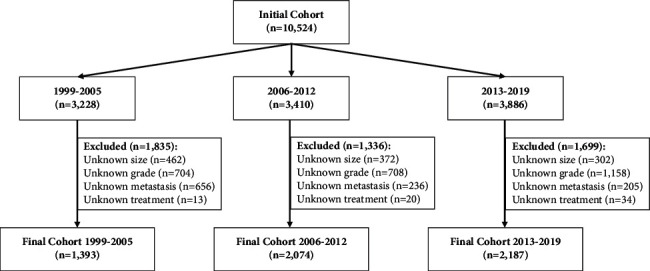
CONSORT diagram of included and excluded patients, stratified by time period.

Of the 10,524 patients identified, 5,654 were included in the final cohort (Figure 1). Patients were excluded for incomplete data on tumor size (*n* = 1,136), tumor grade (*n* = 2,570), metastasis (*n* = 1,097), or treatment (*n* = 67). Importantly, the exclusion rate was similar across all three time periods.

Most patients with ESTS were Caucasian (76.6%), male (55.1%), and ≥60 years of age (47.8%). ESTS occurred predominantly in the lower extremities (76.1%) and was frequently >5 cm in size (69.9%), grade III (58.2%), and without metastasis (77.9%) at diagnosis. The most common histologic subtypes were lipomatous (28.7%) and fibromatous (22.9%).

**Figure 2:**
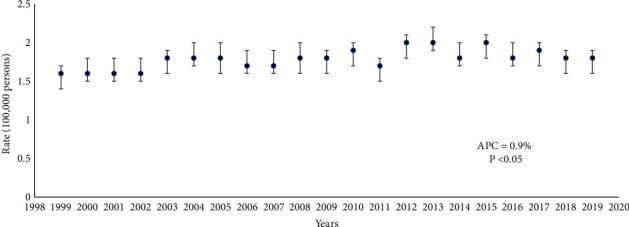
Age-adjusted incidence of ESTS in the United States from 1999 to 2019.

The incidence of ESTS increased from 1.6/100,000 in 1999 to 1.8/100,000 in 2019, representing a 0.9% annual percent change (Figure 2).

In addition, there were substantial changes in patient demographics and clinical characteristics over the study period (Table 1).

For instance, from the earliest to the most recent time period, the proportion of patients ≥60 years of age increased (from 42.9% to 51.9%, *p* < 0.001), as did the proportion of Hispanic patients (from 14.7% to 18.0%, *p*=0.038). Clinically, there was a significant increase in the proportion of grade I tumors (from 17.4% to 20.7%, *p*=0.023). Additionally, there was a significant decrease in the proportion of patients with metastasis (from 28.9% to 13.0%, *p* < 0.001).

Furthermore, there was a shift in histology, with the proportion of ESTS classified as “fibromatous” decreasing from 32.4% to 16.7% in the most recent period, while “other” subtypes increased from 10.1 to 31.1% (*p* < 0.001). Initial treatment with chemotherapy (from 20.2% to 18.4%; *p*=0.002) and surgery (from 96.2% to 92.9%; *p* < 0.001) both decreased over the study period, while radiation remained relatively constant (from 58.6% to 56.1%; *p*=0.372). Patients treated with chemotherapy had a significantly higher proportion of grade III tumors (86.6% vs. 51.0%; *p* < 0.001) >10 cm in size (51.2% vs. 35.6%; *p* < 0.001) and with metastasis (39.3% vs. 17.7%; *p* < 0.001) than those who did not receive chemotherapy. Likewise, patients treated without surgery had a significantly higher proportion of grade III tumors (77.5% vs. 57.2%; *p* < 0.001) >10 cm in size (63.2% vs. 37.4%; *p* < 0.001) and with metastasis (50.5% vs. 20.5%; *p* < 0.001) compared to those who were treated with surgery.

Univariate analyses revealed that several patient and clinical factors were associated with survival in all time periods (Table 2). Older age, larger tumors, higher grade, presence of metastasis, and treatment with chemotherapy were all associated with significantly lower 5-year overall survival. Treatment with surgery was significantly correlated with improved 5-year overall survival.

**Figure 3:**
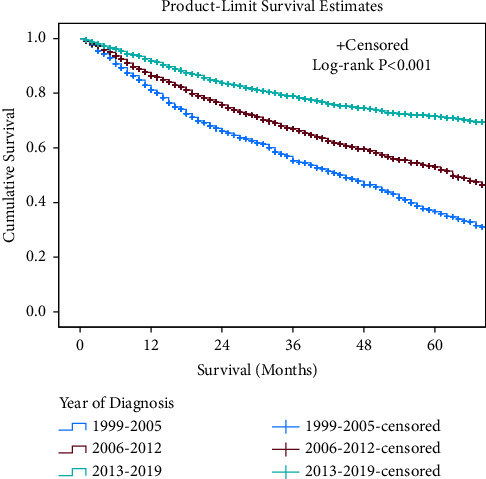
Kaplan–Meier product-limit curves for survival rate of ESTS from 1999 to 2019.

The 5-year overall survival rate progressively improved, 47% in 1999–2005 to 61% in 2006–2012, and finally to 77% in 2013–2019 (Figure 3).

Similarly, there was a stepwise decline in mortality rate from 1999 to 2005 (HR 3.4; 95% CI 3.0–3.8) to 2006–2012 (HR 2.1; 95% CI 1.9–2.3), with the 2013–2019 group having the best overall survival despite adjusting for multiple patient and clinical factors in multivariate analysis (Table 3).

Independent negative predictors of survival included age >30 (HR from 1.9 to 3.9, *p* < 0.001), tumor size 5–10 or >10 cm (HR1.3 to 2.5, *p* < 0.001), grade II or III (HR from 1.9 to 3.3, *p* < 0.001), and metastasis (HR 1.4, *p* < 0.001). Initial treatment with chemotherapy did not demonstrate statistical significance as a negative prognostic variable in this analysis (HR 1.0, *p*=0.444). Positive predictive factors for survival included fibromatous (HR 0.76, *p*=0.002) or lipomatous histologic subtypes (HR 0.55, *p* < 0.001), and treatment with surgery (HR 0.28, *p* < 0.001) or radiation (HR 0.36, *p* < 0.001).

## 4. Discussion

In this population-based study, the incidence of ESTS increased slightly over the 20-year study period, from 1.6/100,000 in 1999 to 1.8/100,000 in 2019. The clinical picture of ESTS also changed, with a greater proportion of older patients diagnosed lower-grade tumors without metastasis. Likewise, the 5-year overall survival rate for ESTS progressively increased from 47% in the 1999–2005 group to 77% in the 2013–2019 group.

Given its bimodal age distribution coupled with the aging population, both the marginal increase in incidence as well as larger proportion of older patients with ESTS is not unexpected [8]. The increase in the proportion of patients with lower-grade tumors at diagnosis may be related to advances in imaging, detection, and histopathologic characterization over the study period [7, 9]. However, the significant decline in the proportion of patients with metastasis, particularly in the most recent 2013 to 2019 period, observed in this study contrasts with the increase reported by Jacobs et al. a decade ago [7]. This discrepancy may be due to differences in study design, as patients with incomplete clinical data on metastasis were excluded from the current work but were included in the original study, potentially skewing calculated proportions and subsequent analyses. Furthermore, it is possible that a declining metastasis rate in recent time periods may be reflective of improved treatment and perhaps earlier detection [10, 11]. Finally, there were changes in histologic subtype of ESTS over time, with an increase in the frequency of “not otherwise specified” and “other” subtypes. This finding is likely related to the discovery of new, rare subtypes of soft tissue sarcomas as well as the reclassification of other subtypes (i.e., malignant fibrous histiocytoma to undifferentiated pleiomorphic sarcoma) [12].

Trends in treatment remained relatively constant over the study period, with surgery and radiation used in 93%–96% and 56%–59% of cases, respectively. Both surgery and radiation were independent positive predictors of survival, consistent with prior findings by Jacobs et al. [7]. Though it is well established that surgical resection reduces rates of metastasis and improves survival, and that radiation reduces the rates of local recurrence, the effect of radiation on overall survival remains inconclusive [13, 14]. New radiation techniques have been developed over the study period, including proton beam therapy, and their effects on local disease control and overall survival remain to be seen [15]. Finally, chemotherapy use slightly declined (23% to 18%) and was negatively correlated with survival. Significant selection bias is present for chemotherapy as its use was largely reserved for the treatment of large, high-grade tumors and metastatic disease in this study. Of note, the SEER database only includes initial treatment course, which may under-represent the utilization of chemotherapy overall. The 5-year survival rate for this subset of patients is very poor at 5–15%, and this confounding likely accounts for chemotherapy as a negative prognostic factor [14, 16].

Interestingly, the proportion of patients treated with surgery declined slightly over the study period. This remains a relatively small subset, though this finding is somewhat counterintuitive in light of the decrease in the percentage of patients presenting with metastatic disease. While these findings may represent a measured approach of attempted systemic therapy as a primary mode of treatment in patients with metastatic disease, further investigation is warranted for this subpopulation.

Multiple predictors of survival were identified in multivariate regression, including older age, tumor size, grade, metastasis, surgery, radiation, and chemotherapy. These prognostic factors are consistent with those identified in previous studies [5, 7, 17]. While the multivariable regression does demonstrate that metastasis at presentation is a significant predictor of 5-year overall survival, the magnitude of the HR is smaller than we would have predicted. The prior analysis by Jacobs et al. yielded an HR of 3.3, while the current study suggests an HR of 1.4. This may be reflective of an improvement in the duration of survival with distant metastases over the study period. The SEER database limits our ability to further understand the complex relationship between the identification of metastasis at presentation, the development of metastasis on surveillance, and how those are impacting and impacted by the various other prognostic factors and treatment modalities.

This study has several limitations. The SEER database lacks clinical data on patient comorbidities; local recurrence rates; and detailed treatment information, including surgical margins, radiation dose/duration, and chemotherapy regimen, precluding analysis of the effects of these factors on survival. Clinical data also remain incomplete for a nontrivial number of patients in each time period, resulting in the exclusion of those patients from our analyses. Although it is possible that patients with incomplete data differed substantially from those with complete data, resulting in overstated survival rates, this is unlikely, as similar absolute and relative trends were noted in prior studies that included patients even with incomplete clinical data.

In summary, this study adds an additional decade's worth of data to prior work by Jacobs et al. and provides an important update on the clinical characteristics and survival rates of ESTS [7]. Although our findings are largely on par with those of the previous study, slight differences in results are likely attributable to extraction of data from 12 registries rather than the original 18 registries. Also, our inclusion criteria were stricter, as all patients with missing clinical data were excluded.

## 5. Conclusions

The 5-year survival rate for ESTS progressively improved over the 20-year study period, with an increasing proportion of older patients diagnosed with lower-grade tumors without metastasis. These findings may be related to earlier detection or more effective treatment over the study period.

## Figures and Tables

**Table 1:** Patient demographics and clinical characteristics stratified by time period.

	*N* = 5,654			*P*
	1999–2005 (*n* = 1,393) *n* (%)	2006–2012 (*n* = 2,074) *n* (%)	2013–2019 (*n* = 2,187) *n* (%)	
*Demographics*				
Sex				
Male	771 (55.3)	1,155 (55.7)	1,190 (54.5)	0.689
Female	622 (44.7)	919 (44.3)	997 (45.5)	
Age group (years)				
<30	111 (8.0)	147 (7.1)	161 (7.4)	<0.001
30–59	684 (49.1)	957 (46.1)	892 (40.8)	
≥60	598 (42.9)	970 (46.8)	1,134 (51.9)	
Race				
Caucasian	1,088 (78.1)	1,597 (77.0)	1,648 (75.4)	0.028
African American	135 (9.7)	195 (9.4)	202 (9.2)	
Other	162 (11.6)	276 (13.3)	314 (14.3)	
Unknown	8 (0.6)	7 (0.3)	23 (1.1)	
Ethnicity				
Non-Hispanic	1,188 (85.3)	1,732 (83.5)	1,794 (82.0)	0.038
Hispanic	205 (14.7)	342 (16.5)	393 (18.0)	
Marital status				
Married	792 (56.9)	1,167 (56.3)	1,247 (57.0)	0.130
Single	283 (20.3)	440 (21.2)	487 (22.2)	
Other	279 (20.0)	381 (18.4)	374 (17.1)	
Unknown	39 (2.8)	86 (4.1)	79 (3.6)	

*Tumor characteristics*				
Primary site				
Upper extremity	343 (24.6)	487 (23.5)	521 (23.8)	0.738
Lower extremity	1,050 (75.4)	1,587 (76.5)	1,666 (76.2)	
Tumor size (cm)				
<5	442 (31.7)	630 (30.4)	630 (28.8)	0.350
5–10	434 (31.2)	631 (30.4)	694 (31.7)	
>10	517 (37.1)	813 (39.2)	863 (39.5)	
Grade				
I	243 (17.4)	434 (20.9)	453 (20.7)	0.023
II	334 (24.0)	422 (20.3)	473 (21.6)	
III	816 (58.6)	1,218 (58.7)	1,261 (57.7)	
Metastasis				
No	990 (71.1)	1,512 (72.9)	1,902 (87.0)	<0.001
Yes	403 (28.9)	562 (27.1)	285 (13.0)	
Histology				
Fibromatous	452 (32.4)	475 (22.9)	365 (16.7)	<0.001
Myxomatous	37 (2.7)	49 (2.4)	57 (2.6)	
Lipomatous	412 (29.6)	609 (29.4)	599 (27.4)	
Myomatous	180 (12.9)	269 (13.0)	276 (12.6)	
Synovial	97 (7.0)	122 (5.9)	103 (4.7)	
NOS	74 (5.3)	151 (7.3)	107 (4.9)	
All other types	141 (10.1)	399 (19.2)	680 (31.1)	

*Treatment*				
Surgery				
No	53 (3.8)	98 (4.7)	156 (7.1)	<0.001
Yes	1,340 (96.2)	1,976 (95.3)	2,031 (92.9)	
Radiation				
No/unknown	577 (41.4)	900 (43.4)	959 (43.9)	0.337
Yes	816 (58.6)	1,174 (56.6)	1,228 (56.1)	
Chemotherapy				
No/Unknown	1,112 (79.8)	1,602 (77.2)	1,784 (81.6)	0.002
Yes	281 (20.2)	472 (22.8)	403 (18.4)	

NOS, not otherwise specified.

**Table 2:** Univariate 5-year survival analysis stratified by time period.

Variable	1999–2005 (*n* = 1,393)		2006–2012 (*n* = 2,074)		2013–2019 (*n* = 2,187)	
	Survival (%)	*P*	Survival (%)	*P*	Survival (%)	*P*
*Demographics*						
Sex						
Male	45	0.136	60	0.249	76	0.057
Female	50		62		79	
Age group (years)						
<30	78	**<0.001**	72	**<0.001**	89	**<0.001**
30–59	62		73		84	
≥60	25		48		71	
Race						
Caucasian	47	0.342	62	0.257	78	0.173
African American	43		57		72	
Other	48		59		79	
Unknown	88		86		83	
Ethnicity						
Non-Hispanic	46	**0.008**	60	0.056	77	0.126
Hispanic	58		67		81	
Marital status						
Married	49	**<0.001**	65	**<0.001**	81	**<0.001**
Single	55		62		79	
Other	32		46		64	
Unknown	67		62		82	

*Tumor characteristics*						
Primary site						
Upper extremity	54	0.151	61	0.185	76	0.226
Lower extremity	45		61		78	
Size (cm)						
<5	60	**<0.001**	73	**<0.001**	89	**<0.001**
5–10	47		60		79	
>10	37		52.2		68	
Grade						
I	65	**<0.001**	87	**<0.001**	93	**<0.001**
II	61		74		86	
III	36		47		68	
Metastasis						
No	53	**<0.001**	68	**<0.001**	82	**<0.001**
Yes	33		43		49	
Histology						
Fibromatous	41	**<0.001**	60	**<0.001**	81	**<0.001**
Myxomatous	46		69		81	
Lipomatous	60		78		89	
Myomatous	43		53		72	
Synovial	54		62		81	
NOS	38		44		58	
All other types	38		47		70	

*Treatments*						
Surgery						
No	21	**<0.001**	14	**<0.001**	35	**<0.001**
Yes	48		63		81	
Radiation						
No/unknown	48	0.731	65	**0.005**	78	0.333
Yes	47		58		77	
Chemotherapy						
No/unknown	49	**0.011**	66	**<0.001**	81	**<0.001**
Yes	40		45		62	
Overall survival (%)	**47**		**61**		**78**	

NOS, not otherwise specified; significant values are in bold.

**Table 3:** Multivariate 5-year survival analysis.

Variable^a^	Hazard ratio (95% CI)	*P*	Total number	Number of events	Median survival time (months)
Period of diagnosis					
2013–2019	Referent		2,187	493	NA^b^
2006–2012	2.1 (1.9, 2.3)	<0.001	2,074	809	63.0 (59.1, 66.0)
1999–2005	3.4 (3.0, 3.8)	<0.001	1,393	734	45.0 (40.8, 49.2)
Age group (years)					
<30	Referent		419	82	NA^b^
30–59	1.9 (1.5, 2.4)	<0.001	2,533	666	NA^b^
≥60	3.9 (3.0, 5.0)	<0.001	2,702	1,288	51.0 (47.7, 54.3)
Marital status					
Married	Referent		3,206	1,049	NA^b^
Single	1.3 (1.2, 1.5)	<0.001	1,210	398	NA^b^
Other	1.3 (1.1, 1.4)	<0.001	1,034	529	45.0 (40.2, 49.8)
Tumor size (cm)					
<5	Referent		1,702	413	NA^b^
5–10	1.5 (1.3, 1.7)	<0.001	1,759	631	70.0 (67.5, 72.5)
>10	2.5 (2.2, 2.8)	<0.001	2,193	992	52.0 (47.3, 50.3)
Grade					
I	Referent		1,130	173	NA^b^
II	1.9 (1.5, 2.3)	<0.001	1,229	305	NA^b^
III	3.3 (2.8, 4.0)	<0.001	3,295	1,558	47.0 (43.7, 50.3)
Metastasis					
No	Referent		4,404	1,299	NA^b^
Yes	1.4 (1.3, 1.6)	<0.001	1,250	727	33.0 (29.1, 36.9)
Histology					
NOS	Referent		332	175	43.0 (32.7, 53.3)
Fibromatous	0.76 (0.64, 0.91)	0.002	1,292	527	63.0 (58.1, 67.9)
Myxomatous	0.76 (0.55, 1.1)	0.099	143	46	72.0 (60.3, 83.7)
Lipomatous	0.55 (0.45, 0.67)	<0.001	1,620	366	NA^b^
Myomatous	0.99 (0.82, 1.2)	0.941	725	306	56.0 (48.1, 63.9)
Synovial	0.95 (0.74, 1.2)	0.665	322	112	NA^b^
All other types	1.1 (0.88, 1.3)	0.564	1,220	504	54.0 (46.8, 61.2)
Surgery					
No	Referent		307	228	10.0 (7.8, 12.2)
Yes	0.28 (0.24, 0.33)	<0.001	5,347	1,808	72.0 (70.5, 73.5)
Radiation					
No	Referent		2,436	822	NA^b^
Yes	0.73 (0.66, 0.80)	<0.001	3,218	1,214	65.0 (62.4, 67.6)
Chemotherapy					
No	Referent		4,498	1,455	NA^b^
Yes	1.0 (0.93, 1.2)	0.444	1,156	581	40.0 (35.7, 44.3)

^a^Primary site, race, ethnicity, and sex were not included in multivariate Cox proportional hazard analysis because variables were not statistically significant on univariate analyses. CI, confidence interval; NOS, not otherwise specified. ^b^Unable to calculate due to >50% survival.

## Data Availability

All data are publicly available through the Surveillance, Epidemiology, and End Results (SEER) database.
